# Metabolic and Transcriptional Adaptations Improve Physical Performance of Zebrafish

**DOI:** 10.3390/antiox10101581

**Published:** 2021-10-07

**Authors:** Franziska J. Heinkele, Bowen Lou, Vanessa Erben, Katrin Bennewitz, Gernot Poschet, Carsten Sticht, Jens Kroll

**Affiliations:** 1 Department of Vascular Biology and Tumor Angiogenesis, European Center for Angioscience (ECAS), Medical Faculty Mannheim, Heidelberg University, D-68167 Mannheim, Germany; F.Heinkele@stud.uni-heidelberg.de (F.J.H.); Bowen.Lou@medma.uni-heidelberg.de (B.L.); Vanessa.Erben@medma.uni-heidelberg.de (V.E.); Katrin.Bennewitz@medma.uni-heidelberg.de (K.B.); 2 Metabolomics Core Technology Platform, Centre for Organismal Studies, Heidelberg University, D-69120 Heidelberg, Germany; gernot.poschet@cos.uni-heidelberg.de; 3 NGS Core Facility, Medical Faculty Mannheim, Heidelberg University, D-68167 Mannheim, Germany; carsten.sticht@medma.uni-heidelberg.de

**Keywords:** danio rerio, diabetes, physical activity, blood glucose, metabolomics, transcriptional profile

## Abstract

Obesity is a worldwide public health problem with increasing prevalence and affects 80% of diabetes mellitus type 2 cases. Zebrafish (*Danio rerio*) is an established model organism for studying obesity and diabetes including diabetic microvascular complications. We aimed to determine whether physical activity is an appropriate tool to examine training effects in zebrafish and to analyse metabolic and transcriptional processes in trained zebrafish. A 2- and 8-week experimental training phase protocol with adult zebrafish in a swim tunnel system was established. We examined zebrafish basic characteristics before and after training such as body weight, body length and maximum speed and considered overfeeding as an additional parameter in the 8-weeks training protocol. Ultimately, the effects of training and overfeeding on blood glucose, muscle core metabolism and liver gene expression using RNA-Seq were investigated. Zebrafish maximum speed was correlated with body length and was significantly increased after 2 weeks of training. Maximum swim speed further increased after 8 weeks of training in both the normal-fed and the overfed groups, but training was found not to be sufficient in preventing weight gain in overfed fish. Metabolome and transcriptome profiling in trained fish exhibited increased blood glucose levels in the short-term and upregulated energy supply pathways as well as response to oxidative stress in the long-term. In conclusion, swim training is a valuable tool to study the effects of physical activity in zebrafish, which is accompanied by metabolic and transcriptional adaptations.

## 1. Introduction

Obesity is a worldwide public health problem with increasing prevalence since 1980 [1], affecting 23.6% of adults in Germany in 2015 [2]. It can result from an over-caloric diet [3] and a lack of physical activity [4] and is associated with severe health problems, such as cardiovascular diseases, non-alcoholic fatty liver disease and diabetes [5,6]. Obesity even affects 80% of diabetes mellitus type 2 patients [7]. Those comorbidities underline the importance to reduce the high obesity prevalence. Body weight is strongly associated with physical activity in humans [8] and acting against obesity is less effective in producing weight loss than in preventing weight gain [9]. Physical exercise is generally considered as a beneficial factor in preventing and improving metabolic diseases [10] and metabolic alterations are also associated with aging, as well as cardiovascular and cognitive impairments [11]. The risk for cardiovascular complications, the leading cause of death globally [12], has been observed to decrease in regularly exercising humans [13]. In addition, physical activity was linked to diminished cognitive decline and a lower prevalence of dementia [14], indicating that physical activity can delay aging via several pathways.

Moreover, physical exercise has been associated with an imbalance of the oxidation-reduction (redox) system in favor of oxidants [15]. Oxidative stress causes oxidative damage of proteins, lipids and nucleic acids and is involved in the pathogenesis of various diseases such as metabolic syndrome, indicating that maintenance of redox balance is crucial for health. [16]. However, exercise-induced production of free radicals and reactive oxygen species (ROS) can also have beneficial effects on cellular signaling and immune reactions [15].

Animal models enable researchers to investigate the effects of physical activity on the metabolism and transcriptome. Zebrafish (*Danio rerio*) is an established model organism for studying obesity and diabetes including diabetic microvascular complications [17,18]. Although zebrafish has intensively been studied recently in different metabolic diseases, it is not established as a model to perform exercise training with. Physical activity can be mimicked with zebrafish trained in a swim tunnel system. The swim tunnel is based on a propeller, which generates a water flow in a swim tube, forcing the zebrafish to swim against the current. However, the effects of physical performance on physiological parameters and the zebrafish metabolism and transcriptome have rarely been studied so far [19,20,21,22,23].

We aimed to determine whether the swim tunnel system is an appropriate tool to achieve training effects within a reasonable period of time and whether training can prevent weight gain in overfed zebrafish. Subsequently, we analysed how the metabolome and transcriptome responded adapted to the training procedure. Our data show that swim training enhanced the physical performance of zebrafish, and that metabolic and transcriptomic adaptations underlie the physical improvements.

## 2. Materials and Methods

### 2.1. Fish Maintenance

Zebrafish of the ABTL strain were reared and maintained at 28 °C and kept under 13 h light/11 h dark cycle in groups of 6 fish per tank. Zebrafish with an age of 6 months and older were considered as adults.

### 2.2. Swim Tunnel

The exercise experiments were performed in a 170 mL (230 V/50 Hz; model: SW10000) swim tunnel system of Loligo^®^ Systems (Viborg, Denmark). According to Wakamatsu et al., who performed a study with the same swim tunnel, water velocity is linear to the voltage applied with velocity being around 30 cm/sec at 2.5 V and increasing by approximately 13 cm/sec per Volt [24]. Therefore, speed can be estimated from the applied voltage [21]; however, the study mainly focused on the relative improvement of zebrafish swim performance rather than on the absolute speed.

### 2.3. Experiment Setup

**Figure 1:**
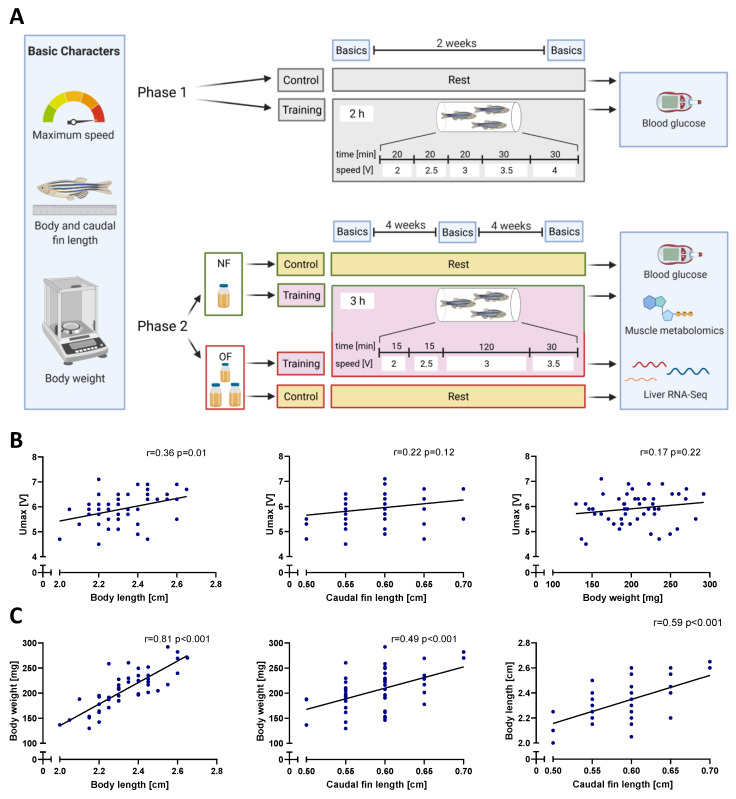
Experiment setup and initial basic character correlations. (**A**): The experiment was split into two phases. Phase 1: 2-week training; Phase 2: 8-week training including overfeeding. Training protocols differed in current velocity and total duration. Upon completion of the phases, fish were sacrificed, blood glucose was measured, and in phase 2 experiment metabolomics and RNA-Seq data were determined. NF: normal feeding, OF: overfeeding. (**B**): positive training-independent correlations of body length, caudal fin length and body weight with the maximum speed (Umax). (**C**): Positive correlations between zebrafish body length, body weight and caudal fin length. Simple linear regression lines were fitted. N = 50 fish per correlation analysis, r: Pearson correlation coefficient, *p*: two-tailed *p* value.

The phase 1 experiment (Figure 1A) was performed over a period of 2 weeks (5 sets of trainings in total + 1 additional training directly before sacrifice) and consisted of a non-trained control group and a trained group, each comprising 12 fish. The phase 2 experiment (Figure 1A), in which the training period was extended to 8 weeks (20 sets of trainings in total), was performed with a normal-fed non-trained group (NF-control), a normal-fed trained group (NF-training), an overfed non-trained group (OF-control) and an overfed trained group (OF-training) including 6 fish each. All experiments applied adult ABTL zebrafish and equal numbers of females and males in each group to avoid different group conditions. Fish of all training groups were trained every other day from Monday to Friday indicating alternating twice or thrice per week in phase 1 and phase 2.

Upon completion of the 2-week or 8-week training phase, respectively, zebrafish were fasted overnight and sacrificed the next morning. Fish sacrifice served for blood glucose measurement, collection of muscles for determination of glycogen content and metabolomics data and collection of livers for RNA-sequencing (RNA-Seq) data acquisition.

### 2.4. Feeding

In the phase 1 experiment, fish were fed with 1 mL of living shrimps per fish in the morning and 1 spoon of SDS 400 (Special Diets Service, Essex, UK) per tank in the afternoon. In the phase 2 experiment, the normal-fed (NF) groups received 0.75 mL of living shrimps per fish in the morning and 2 spoons of SDS 400 per tank in the afternoon. The overfed (OF) groups were fed with 9 mL of living shrimps per fish spread over the morning (3 times 3 mL) and 2 spoons of SDS 400 per tank in the afternoon. For training groups, a digestive break of minimum 2 h was considered between feeding and training.

### 2.5. Determination of Basic Characters

Measurement of the basic characters included measurement of body length, body weight, caudal fin length, and the maximum speed of each fish. For the phase 1 experiment, parameters were recorded before and after the 2-week training phase. In the phase 2 experiment basic measurements were performed at the beginning and after 4 weeks and 8 weeks of training. To ensure equal initial strength of all groups, group building was carried out after the collection of initial basic characters with no statistically significant differences between groups. For the measurement of body length, body weight and caudal fin length, fish were anaesthetised in a tank with 160 mg/L tricaine for 2 min. Body length and caudal fin length were determined with a ruler and the weight with an analytical balance (ABS-N_ABJ-NM_ACS_ACJ, Kern & Sohn, Puchheim, Germany). The treatment with tricaine was performed after the maximum speed measurement to avoid adverse effects on the zebrafish swimming capability.

For the measurement of the maximum speed, fish were placed in the swim tunnel separately and the velocity was increased incrementally. The initial voltage (2 V) was increased by 0.5 V every 30 s until 4.5 V, followed by an increase by 0.2 V every 20 s until the fish was not able to keep swimming and fell back into the mesh.

### 2.6. Training Protocol

Training groups were trained from Monday to Friday every other day in groups of three fish. The weekends served as recovery time for the training groups. Swim protocols are summarised in Figure 1A. Protocol 1 was applied in the phase 1 experiment and comprised the following steps: The voltage was increased from 2 V to 2.5 V and 3 V, each step taking 20 min. Next, 30 min at each 3.5 V and 4 V completed the training, which had a total duration of 2 h. Protocol 2 was applied in the phase 2 experiment. Training sessions started at 2 V followed by 2.5 V, both for 15 min. Subsequently, the voltage was increased to 3 V for 120 min and further to 3.5 V for 30 min, extending the training to a total of 3 h. Thus, protocol 2 comprised a longer time span whereas protocol 1 had a higher average swim speed. In the phase 1 experiment, training groups received the last training session directly before they were sacrificed to have the maximum effect of the training on blood glucose levels. In the phase 2 experiment, fish were sacrificed 24 h after their last training session to explore long-term training effects.

### 2.7. Determination of Blood Glucose

Fasting blood glucose was measured with a FreeStyle Freedom Lite–blood glucose monitoring system (Abbott, Wiesbaden, Germany), directly after fish were sacrificed.

### 2.8. Glycogen Measurement

Glycogen assay was performed with zebrafish muscle tissue using the Glycogen Assay Kit from Abcam (Cambridge, UK) according to the manufacturer’s instructions.

### 2.9. Metabolomics Profile

Detection was performed with the Metabolomics Core Technology Platform from the Centre of Organismal Studies Heidelberg. After fish were sacrificed, muscle tissue was collected from the phase 2 experiment for metabolite analyses of adenosine compounds and free amino acids as described previously [25].

### 2.10. Statistics

Experimental results are shown as linear regressions lines for correlation analyses and box whisker plots with the median of the groups showing all single measurement points from minimum to maximum. Statistical significance between different groups was analysed using Student’s *t*-test for phase 1 experiment and one-way ANOVA or Kruskal-Wallis test for phase 2 experiment. GraphPad Prism 9.1.1 (GraphPad Software Inc, San Diego, CA, USA) was used for analyses of maximum speed, body weight and metabolomics and *p* values below 0.05 were considered as significant: * *p* < 0.05, ** *p* < 0.01, *** *p* < 0.001.

### 2.11. RNA-Seq Analysis

RNA was isolated from liver samples from fish of the phase 2 experiment using the RNeasy^®^ Mini Kit from QIAGEN (Hilden, Germany). Library construction and sequencing were performed with BGISEQ-500 (BGI, Hong Kong, China). Most of the procedure was conducted with R and Bioconductor using the NGS analysis package systempipeR [26]. Quality control of raw sequencing reads was performed using FastQC (Babraham Bioinformatics). Low-quality reads were removed using trim_galore (version 0.6.4). The resulting reads were aligned to zebrafish genome version GRCz11 from NCBI and counted using Kallisto version 0.46.1 [27]. The count data was transformed to log2-counts per million (logCPM) using the voom-function from the limma package [28]. The GSEA pathway analysis was made with the ClusterProfiler package [29] in R using the pathway information from org.Dr.eg.db [30]. Barplots were created using the ggplot2 package (version 3.3.5).

## 3. Results

### 3.1. Maximum Speed Correlated with Body Length

Determination of maximum swim speed is a good measure for zebrafish swimming capability but is influenced by various factors [24]. Thus, we first analysed how baseline parameters including body length, caudal fin length and body weight determine the swimming ability of zebrafish. The parameters were measured at the beginning of all experiments for each fish independent of the experimental setup (Figure 1A). It was found that zebrafish maximum speed (Umax) was positively correlated with body length whereas caudal fin length and body weight were not-significantly correlated (Figure 1B); yet, body weight, body length and caudal fin length were strongly correlated with each other (Figure 1C). Thus, suitable selection of zebrafish regarding basic characters for swim tunnel experiments is an important consideration.

### 3.2. Swim Training Improves the Physical Performance of Zebrafish

**Figure 2:**
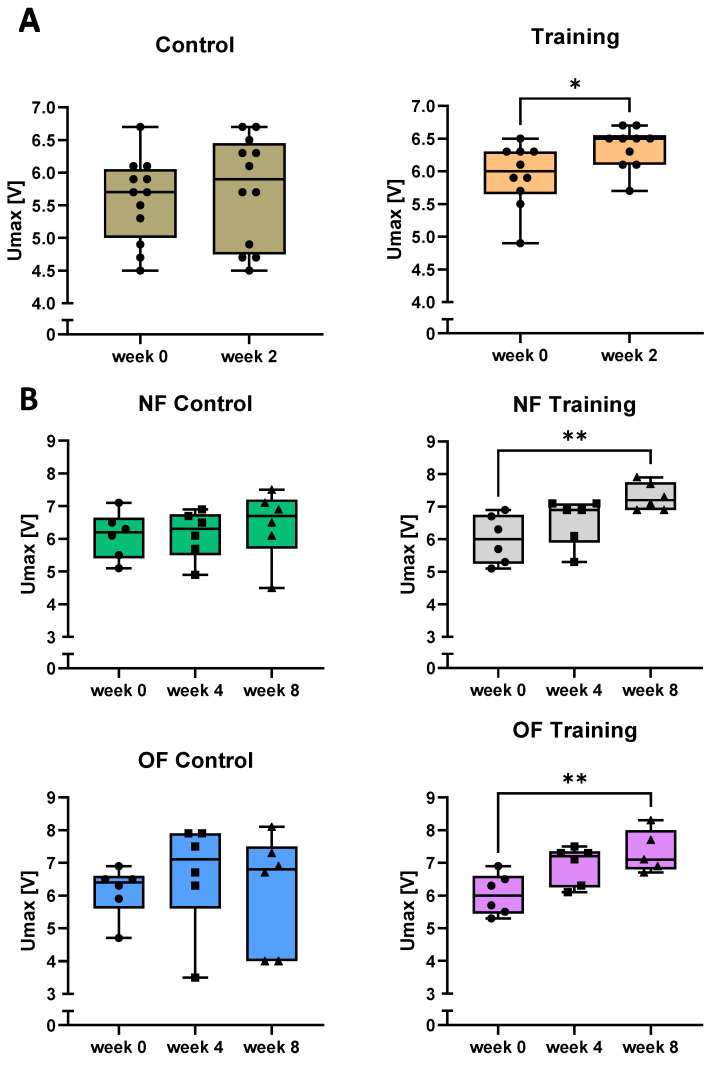
Swim tunnel training improved zebrafish swimming capability. (**A**): phase 1 training increased Umax significantly after 2 weeks of training (*p* = 0.0323). Control: N = 12 fish, Training: N = 10 fish. Two-tailed unpaired *t*-test. (**B**): phase 2 training increased Umax in both NF and OF groups significantly after 8 weeks of training (NF-training *p* = 0.0037, OF-training *p* = 0.0012). N = 6 fish per group. One-way ANOVA test or Kruskal-Wallis test. NF: normal feeding; OF: overfeeding; Umax: maximum speed. * *p* < 0.05, ** *p* < 0.01.

In order to assess the maximum speed of zebrafish after the 2-week training period including standard feeding, maximum speed at the beginning and after 2 weeks of training in comparison to a control group without training was determined (Figure 2A). It was found that the maximum speed of the trained fish increased significantly after 2 weeks of training, while the control group did not show improvement of Umax. Next, we assessed, if an extensive training period over 4 weeks and 8 weeks in combination with an overfeeding diet (Figure 1A), would further increase maximum speed of zebrafish and if the training may prevent a weight gain or if a higher caloric intake may improve swimming performance. For these experiments, we assessed initial Umax before the training exercise (week 0) as well as after 4 weeks and 8 weeks for all groups (Figure 2B). Maximum speed increased time-dependently after 4 weeks of training and significantly after 8 weeks of training in both the NF and the OF groups. The untrained groups, independently from feeding state, exhibited no significant difference. Interestingly, analysing all zebrafish from both experiments together, we observed significantly higher initial maximum speeds of males compared with females (Figure S1).

### 3.3. Swim Training Only Moderately Attenuates Weight Gain in Overfed Zebrafish

**Figure 3:**
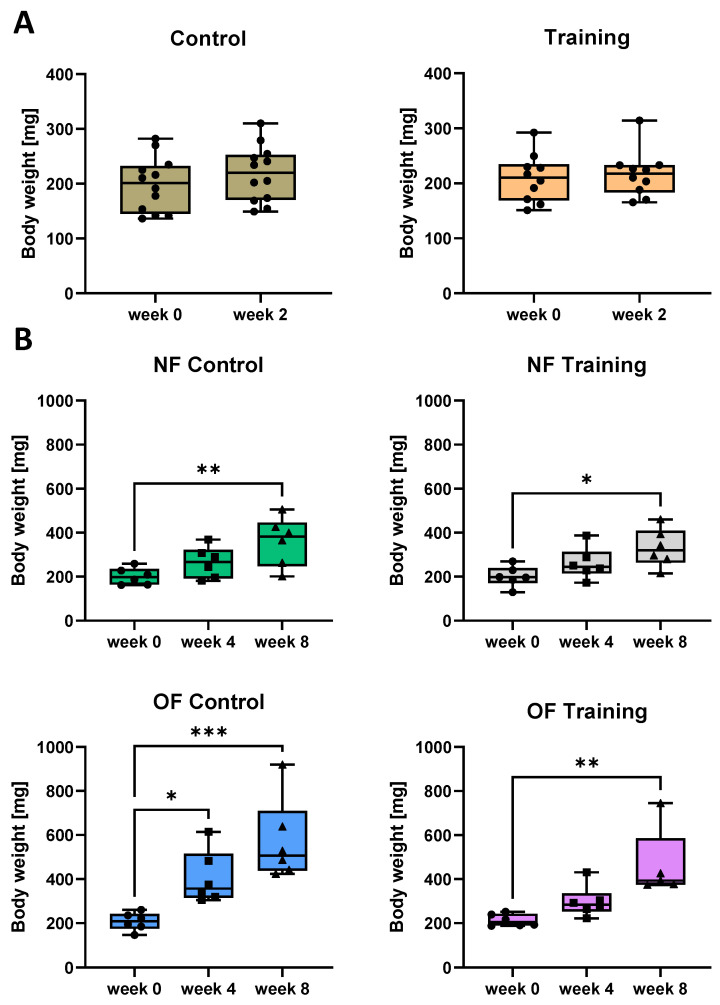
Swim tunnel training prevented weight gain in zebrafish only moderately. (**A**): phase 1 training did not alter zebrafish body weight. Control: N = 12 fish; Training: N = 10 fish. Two-tailed unpaired *t*-test. (**B**): phase 2 training hardly prevented weight gain in normalfed and overfed zebrafish, respectively. Body weight increased after 4 weeks significantly in the OF-control group (*p* = 0.0461). After 8 weeks, body weight was increased in both the NF and OF-control group (*p* = 0.0090 and 0.0006, respectively) and in the training groups (*p* = 0.0161 and 0.0021, respectively). N = 6 fish per group. One-way ANOVA test or Kruskal-Wallis test. NF: normal feeding; OF: overfeeding. * *p* < 0.05, ** *p* < 0.01, *** *p* < 0.001.

To test the suitability of swim training to prevent obesity in overfed zebrafish, we applied the phase 2 experiment consisting of the 8 weeks of training in combination with a high calorie intake. In phase 1 experiment without overfeeding and 2 weeks of training only, however, zebrafish weight was not affected by swimming training since we did not observe significant changes in body weight after 2 weeks in both the control and the training group (Figure 3A).

At the beginning of phase 2 experiment, mean body weight of the zebrafish was around 200 mg (Figure 3B). After 4 weeks, body weight increased slightly in the NF-control group and significantly in the OF-control group without training, while both groups exhibited a significant increase in body weight after 8 weeks (Figure 3B, left). In both the NF-training and OF-training groups, body weight was not significantly increased after 4 weeks but after 8 weeks of training, however, the weight gain was less significant in the OF training group compared with the OF control group. This indicates that swim training of zebrafish could only partially prevent a strong weight gain after high caloric intake (Figure 3B, right).

### 3.4. Swim Training Alters the Metabolome

**Figure 4:**
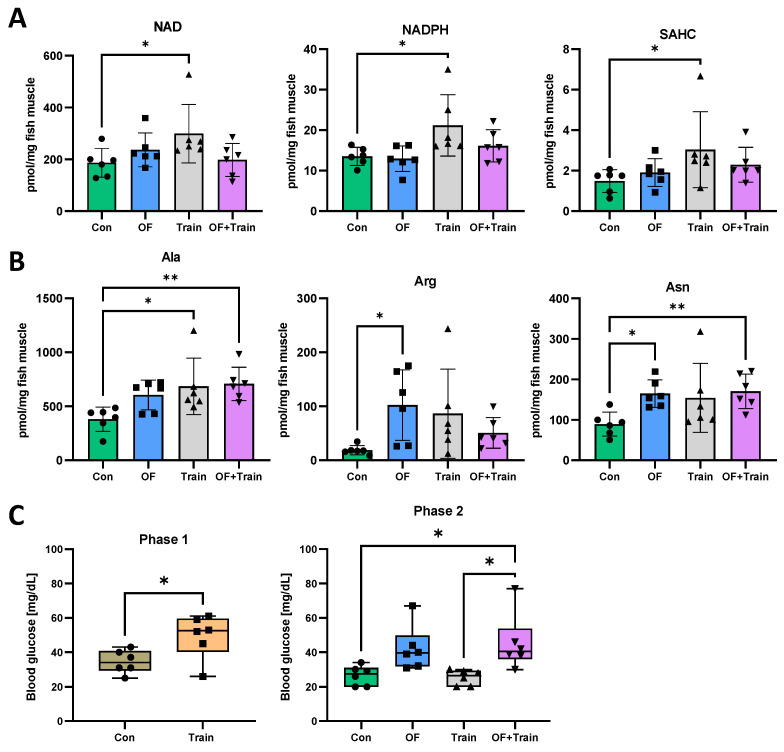
Swim tunnel training significantly altered the metabolome in zebrafish muscle. (**A**): increased concentrations of adenosine levels in trained zebrafish. NAD: nicotinamide dinucleotide, NADPH: nicotinamide dinucleotide phosphate, SAHC: S-adenosyl-homocysteine. N = 6 fish per group. (**B**): Alterations on amino acid levels in trained and overfed zebrafish. Ala: Alanine, Arg: Arginine, Asn: Asparagine. N = 6 fish per group. (**C**): Alterations on fasting blood glucose level. In phase 1 experiment, blood glucose was measured directly after the last training session whereas blood glucose was measured 24 h later in phase 2 experiment. Con: Control; OF: overfeeding; Train: Training. N = 6 fish per group. Statistical tests: Two-tailed unpaired t-test (phase 1) and one way ANOVA test or Kruskal-Wallis test (phase 2). * *p* < 0.05, ** *p* < 0.01.

Thus far we have shown an improved physical performance of zebrafish after 2 weeks and 8 weeks of training (Figure 2) and a moderately reduced weight gain in overfed and exercised zebrafish (Figure 3). In order to analyse the metabolome profile in trained and overfed fish, we assessed several metabolites linked to catabolic pathways in zebrafish muscle tissue after 8 weeks. Thereby, we detected metabolites with significantly different levels in the different groups, such as nicotinamide dinucleotide (NAD), nicotinamide dinucleotide phosphate (NADPH) and S-adenosyl-homocysteine (SAHC) (Figure 4A), the concentration of which was increased in the NF-training group compared with the NF-control group. Furthermore, concentration of the amino acid alanine was increased in the NF-training group compared with the NF-control group and further increased in the OF-training group compared with the NF-control group (Figure 4B). Overfeeding also resulted in higher arginine levels compared with the NF-control group. No significant changes of arginine levels were seen under training conditions. Likewise, asparagine was increased in the OF-control group compared to the NF-control group but as well in the OF-training group compared to the NF-control group (Figure 4B). Interestingly, metabolite levels were almost consistently found to be lowest in the untreated group and to increase by training, overfeeding, or both combined. In addition, muscle glycogen content did not significantly differ between the control and training groups in both experiments (Figure S2). Lastly, we assessed blood glucose after both experimental setups (Figure 4C). After phase 1, blood glucose was measured shortly after the last training after 2 weeks to determine the short-term effects of the training protocol on the glucose metabolism. We found significantly increased blood glucose levels in the training group compared to the control group (Figure 4C). In order to assess glucose homeostasis in long-term effects, the blood glucose level of trained fish was measured 24 h after the last training session in the phase 2 experiment (Figure 4C). We detected higher blood glucose levels in both OF groups and significantly increased levels in the OF-training group compared with the NF-control group and the NF-training group.

### 3.5. Swim Training Alters the Transcriptional Profile Linked to Energy Metabolism and Oxidative Stress

**Figure 5:**
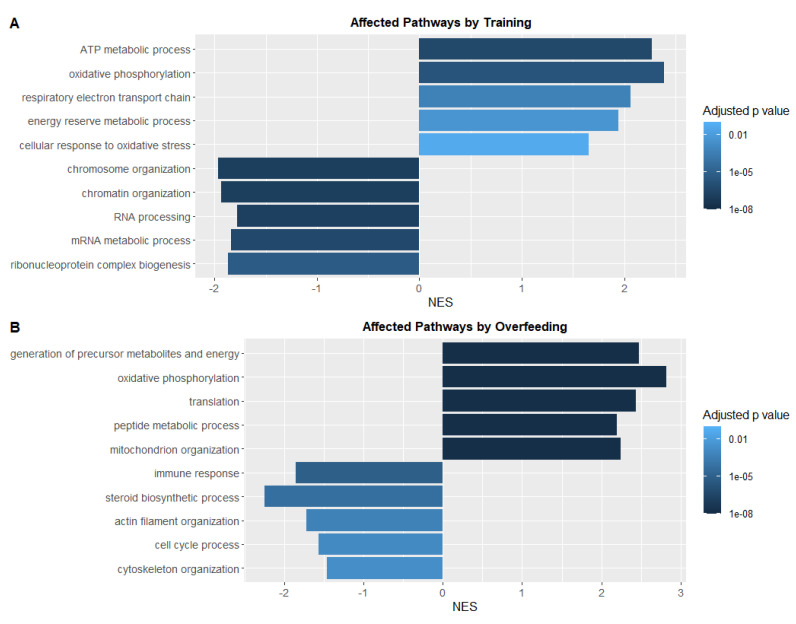
Pathway-Analysis of RNA-Sequencing data in livers showed patterns distinguishing training and overfeeding groups from controls. (**A**): Up- and downregulated pathways in the NF-training compared to the NF-control group. (**B**): Up- and downregulated pathways in the OF-control compared to the NF-control group. Five significantly enriched biological process terms were selected for each category and colored according to adjusted *p* value. NES: normalised enrichment score.

In order to identify transcriptional adaptations linked to physical performance in zebrafish, we performed RNA sequencing in liver tissue. When pathway analysis was performed comparing training effects against the control group, we observed upregulated pathways such as “ATP metabolic process”, “oxidative phosphorylation”, or “cellular response to oxidative stress” (Figure 5A). Due to the increased energy generation, other biological processes were decreased, such as “chromosome organization” or “RNA processing” appeared to be downregulated. “Oxidative phosphorylation” was also upregulated in the OF-control group compared to the NF-control group, as well as “translation” and “mitochondrion organization” (Figure 5B). Downregulated pathways were here more heterogeneous including “immune response” and “cell cycle process” pathways. Together, the data show a metabolic and transcriptional adaptation to the increased physical performance and to increased calorie intake in zebrafish.

## 4. Discussion

In this study we were able to show the effectiveness of physical activity (swim training) on maximum swim speed of zebrafish. We found that zebrafish maximum speed is positively correlated with body length. Swim training improved the performance of the zebrafish in a time-dependent manner whereas body weight gain was only slightly attenuated. In subsequent analyses, the metabolomic and transcriptional profiles displayed adaptational processes affected by training.

To analyse effects of physical activity in zebrafish, we developed two different training protocols. Both were broadly accepted by zebrafish and efficient in increasing the maximum speed after 2 and 8 weeks of training, respectively. Similar results of increasing maximum speed by exercising of zebrafish were recently reported [20]. Additionally, we found a sex-specific difference with males having greater maximum speed than female zebrafish. Other studies only found weak impact on the maximum speed with sex [24]. Since we found additionally large gender differences in gene expression one might consider employing one gender only to eliminate inter-gender variability.

Despite the effectiveness of our protocols in improving physical performance, the feeding protocol requires further adaptation to evaluate more precisely how swim training affects body weight. Nevertheless, as the increase of weight in the training group might be attributable to a gain in muscle mass to some extent, an examination of the body mass composition might further inform about the effectiveness of the swim training in preventing obesity. Endurance training on zebrafish larvae have shown that though both trained and untrained fish had plenty of fat depositions, trained fish were overall heavier with increased cross-sectional red muscle fiber area [22]. Another study found gene expression markers associated with muscle growth to be upregulated after swim training [23], which supports the suggestion of swim training being capable to induce a redistribution of zebrafish body tissue.

Furthermore, physical exercise proved to impact metabolic processes such as glucose homeostasis. The increased blood glucose levels measured directly after training might be explainable by the release of stress hormones such as adrenalin during training that trigger the breakdown of glycogen [31]. The released glucose is required for muscle ATP synthesis during physical activity [32] and thus might help to maintain swimming performance over a longer period. In contrast, a study focusing on the short-term effects of training found glucose and glycogen levels to be depleted at high training intensities due to the consumption of reserves [19]. Consequently, glucose levels might fluctuate greatly during and after training, however, the increased blood glucose we observed was rather a short-term effect that disappeared 24 h later. Experiments addressing how blood glucose of diabetes mutants is affected by swim training might be interesting for future studies. Moreover, we detected increased levels of NAD in trained fish–when converted to NADH, it transfers reducing equivalents from glycolysis and the citric acid cycle and is therefore crucial for energy generation [33]. Training also elevated NADPH, which plays a pivotal role in the antioxidation system. It supplies antioxidants such as glutathione with reducing equivalents and a high cytoplasmic NADPH level is associated with longevity [34]. In addition, NADPH serves as an essential electron source for reductive synthesis of many structures including non-essential amino acids [35]. In accordance with our finding of higher alanine levels in trained fish, others found alanine synthesis to be increased during training needed to fuel the higher energy demands [36,37], while increased amino acid levels in general favor muscle growth [38]. Especially arginine, which regulates vasodilation, has shown to increase during exercise and oral arginine supplementation increases protein synthesis [39]. Even though not significant, we also observed elevated arginine levels in trained zebrafish.

The transcriptomic pathway analysis revealed that physical exercise induces oxidative stress. In accordance, increased oxidative damage markers were detected in humans upon aerobic and anaerobic exercise. While we consider zebrafish to be a valuable tool to study physical exercise, rodent animal models also have been used to study the activity of antioxidative pathways upon physical exercise. Exercise increased oxidative stress levels not only in blood and skeletal muscle but also in many other organs, suggesting exercise-associated oxidative stress induction to affect the whole body. Remarkably, regular exercise was observed to increase the antioxidant capacity, explaining why long-term training reduces acute exercise oxidative stress [15]. Exploring the effects of different training intensities and durations on zebrafish redox balance and the impact of training on remission of diabetic organ complications linked to oxidative stress in zebrafish remains to be investigated. While the swim training model opens the perspective to study and prevent age-related diseases, zebrafish is not an established model for aging due to their lifetime of up to 5 years. In comparison to zebrafish lifetime both swimming protocols were short-term and thus did not modify markers of senescence.

Furthermore, our transcriptomic pathway analysis reflects the increased energy requirement by physical exercise with energy consumption pathways being upregulated. Similar effects were seen in muscles when increased oxidative phosphorylation and ATP production was induced by physical exercise [40]. In accordance with our findings, a systematic review on human muscle adaptations to training showed that proteins associated with the electron transport chain were increased by exercise training [41]. In line with our transcriptomic analysis identifying an upregulated oxidative phosphorylation and mitochondrion organization in overfed zebrafish, another study has postulated that organisms adapt to overfeeding and higher fat intake with increased mitochondrial activity and mass [42].

Limitations of the study are the small number of fish per group, which reduces the expressiveness of our analyses, as well as sex-related differences in maximum speed. Nevertheless, the swim tunnel is a valuable, easily applicable tool that enables imitation of physical activity in zebrafish. It facilitates exploration of training effects not only on metabolomics or genomics in healthy animals, but also on the phenotype of obesity-related diseases such as diabetes. In addition, swim tunnel systems are expandable for the measurement of oxygen consumption during physical activity, which is a significant parameter for sports physiology in humans [43]. The high translational potential of swim training is further highlighted by its flexibility in training intensity and duration, making it easily adaptable to clinical protocols. Ultimately, the swim protocols proposed in this paper can be customized to evaluate zebrafish training effects on disease, therapeutic treatments, regeneration or to evaluate potential roles of specific genes on zebrafish swimming capability.

## 5. Conclusions

The swim tunnel is an effective measure for the maximum speed of zebrafish. Regardless of feeding, swim tunnel training improves time-dependently the swimming performance of zebrafish, which is associated with body length. Moreover, training induces metabolomic and transcriptomic alterations affecting energy consumption pathways. Therefore, zebrafish swimming training can be regarded as a valuable tool for assessing the association of physical activity on disease outcomes.

## Data Availability

The RNA-Seq dataset produced in this study is available at https://www.ncbi.nlm.nih.gov/geo/query/acc.cgi?acc=GSE183023 (last accessed: 28 September 2021).

## Supplementary Materials

The following are available online at https://www.mdpi.com/article/10.3390/antiox10101581/s1, Figure S1: Initial maximum speed was higher in male than in female fish, Figure S2: Glycogen content in zebrafish muscle.

## References

[1] James W.P.T. (2008). The epidemiology of obesity: The size of the problem. J. Intern. Med..

[2] OECD (2017). Overweight and obesity among adults. Health at a Glance 2017.

[3] Romieu I., Dossus L., Barquera S., Blottière H.M., Franks P.W., Gunter M., Hwalla N., Hursting S.D., Leitzmann M., Margetts B. (2017). Energy balance and obesity: What are the main drivers?. Cancer Causes Control.

[4] Lee H.S., Lee J. (2021). Effects of Exercise Interventions on Weight, Body Mass Index, Lean Body Mass and Accumulated Visceral Fat in Overweight and Obese Individuals: A Systematic Review and Meta-Analysis of Randomized Controlled Trials. Int. J. Environ. Res. Public Health.

[5] Sarwar R., Pierce N., Koppe S. (2018). Obesity and nonalcoholic fatty liver disease: Current perspectives. Diabetes Metab. Syndr. Obes..

[6] Scherer P.E., Hill J.A. (2016). Obesity, Diabetes, and Cardiovascular Diseases: A Compendium. Circ. Res..

[7] Bloomgarden Z.T. (2000). American Diabetes Association Annual Meeting, 1999: Diabetes and obesity. Diabetes Care.

[8] Jakicic J.M., Rogers R.J., Davis K.K., Collins K.A. (2018). Role of Physical Activity and Exercise in Treating Patients with Overweight and Obesity. Clin. Chem..

[9] Hill J.O., Wyatt H.R., Peters J.C. (2012). Energy Balance and Obesity. Circulation.

[10] Pedersen B.K., Saltin B. (2015). Exercise as medicine—evidence for prescribing exercise as therapy in 26 different chronic diseases. Scand. J. Med. Sci. Sports.

[11] Garatachea N., Pareja-Galeano H., Sanchis-Gomar F., Santos-Lozano A., Fiuza-Luces C., Morán M., Emanuele E., Joyner M.J., Lucia A. (2015). Exercise attenuates the major hallmarks of aging. Rejuvenation Res..

[12] Cardiovascular Diseases (CVDs). http://www.who.int/mediacentre/factsheets/fs317/en/index.html.

[13] Hannan M., Kringle E., Hwang C.-L., Laddu D. (2021). Behavioral Medicine for Sedentary Behavior, Daily Physical Activity, and Exercise to Prevent Cardiovascular Disease: A Review. Curr. Atheroscler. Rep..

[14] Brown B.M., Peiffer J.J., Martins R.N. (2013). Multiple effects of physical activity on molecular and cognitive signs of brain aging: Can exercise slow neurodegeneration and delay Alzheimer’s disease?. Mol. Psychiatry.

[15] Kawamura T., Muraoka I. (2018). Exercise-Induced Oxidative Stress and the Effects of Antioxidant Intake from a Physiological Viewpoint. Antioxidants.

[16] Marseglia L., Manti S., D’Angelo G., Nicotera A., Parisi E., Di Rosa G., Gitto E., Arrigo T. (2014). Oxidative stress in obesity: A critical component in human diseases. Int. J. Mol. Sci..

[17] Heckler K., Kroll J. (2017). Zebrafish as a Model for the Study of Microvascular Complications of Diabetes and Their Mechanisms. Int. J. Mol. Sci..

[18] Zang L., Maddison L.A., Chen W. (2018). Zebrafish as a Model for Obesity and Diabetes. Front. Cell Dev. Biol..

[19] da Rosa J.G., Barcellos H.H., Idalencio R., Marqueze A., Fagundes M., Rossini M., Variani C., Balbinoti F., Tietböhl T.M., Rosemberg D.B. (2017). Just Keep Swimming: Neuroendocrine, Metabolic, and Behavioral Changes After a Forced Swimming Test in Zebrafish. Zebrafish.

[20] Usui T., Noble D.W.A., O’Dea R.E., Fangmeier M.L., Lagisz M., Hesselson D., Nakagawa S. (2018). The French press: A repeatable and high-throughput approach to exercising zebrafish (Danio rerio). PeerJ.

[21] Wakamatsu Y., Kashima M., Hirata H. (2020). A Reproducible Protocol to Measure the Critical Swimming Speed of Adult Zebrafish. Bio-Protocol.

[22] van der Meulen T., Schipper H., van den Boogaart J.G., Huising M.O., Kranenbarg S., van Leeuwen J.L. (2006). Endurance exercise differentially stimulates heart and axial muscle development in zebrafish (Danio rerio). Am. J. Physiol. Regul. Integr. Comp. Physiol..

[23] Palstra A.P., Tudorache C., Rovira M., Brittijn S.A., Burgerhout E., van den Thillart G.E., Spaink H.P., Planas J.V. (2010). Establishing zebrafish as a novel exercise model: Swimming economy, swimming-enhanced growth and muscle growth marker gene expression. PLoS ONE.

[24] Wakamatsu Y., Ogino K., Hirata H. (2019). Swimming capability of zebrafish is governed by water temperature, caudal fin length and genetic background. Sci. Rep..

[25] Schmöhl F., Peters V., Schmitt C.P., Poschet G., Büttner M., Li X., Weigand T., Poth T., Volk N., Morgenstern J. (2019). CNDP1 knockout in zebrafish alters the amino acid metabolism, restrains weight gain, but does not protect from diabetic complications. Cell Mol. Life Sci..

[26] Backman T.W.H., Girke T. (2016). systemPipeR: NGS workflow and report generation environment. BMC Bioinform..

[27] Bray N.L., Pimentel H., Melsted P., Pachter L. (2016). Near-optimal probabilistic RNA-seq quantification. Nat. Biotechnol..

[28] Ritchie M.E., Phipson B., Wu D., Hu Y., Law C.W., Shi W., Smyth G.K. (2015). limma powers differential expression analyses for RNA-sequencing and microarray studies. Nucleic Acids Res..

[29] Wu T., Hu E., Xu S., Chen M., Guo P., Dai Z., Feng T., Zhou L., Tang W., Zhan L. (2021). clusterProfiler 4.0: A universal enrichment tool for interpreting omics data. Innovation.

[30] Carlson M. org.Dr.eg.db: Genome Wide Annotation for Zebrafish. R Package Version 3.13.0. http://bioconductor.org/packages/release/data/annotation/html/org.Dr.eg.db.html.

[31] Kolnes A.J., Birk J.B., Eilertsen E., Stuenæs J.T., Wojtaszewski J.F., Jensen J. (2015). Epinephrine-stimulated glycogen breakdown activates glycogen synthase and increases insulin-stimulated glucose uptake in epitrochlearis muscles. Am. J. Physiol. Endocrinol. Metab..

[32] De Feo P., Di Loreto C., Lucidi P., Murdolo G., Parlanti N., De Cicco A., Piccioni F., Santeusanio F. (2003). Metabolic response to exercise. J. Endocrinol. Investig..

[33] Verdin E. (2015). NAD⁺ in aging, metabolism, and neurodegeneration. Science.

[34] Murphy M.P. (2012). Mitochondrial thiols in antioxidant protection and redox signaling: Distinct roles for glutathionylation and other thiol modifications. Antioxid. Redox Signal..

[35] Stehling O., Lill R. (2013). The role of mitochondria in cellular iron-sulfur protein biogenesis: Mechanisms, connected processes, and diseases. Cold Spring Harbor Perspect. Biol..

[36] Rennie M.J., Tipton K.D. (2000). Protein and amino acid metabolism during and after exercise and the effects of nutrition. Annu. Rev. Nutr..

[37] Mourtzakis M., Saltin B., Graham T., Pilegaard H. (2006). Carbohydrate metabolism during prolonged exercise and recovery: Interactions between pyruvate dehydrogenase, fatty acids, and amino acids. J. Appl. Physiol. (1985).

[38] Jäger R., Kerksick C.M., Campbell B.I., Cribb P.J., Wells S.D., Skwiat T.M., Purpura M., Ziegenfuss T.N., Ferrando A.A., Arent S.M. (2017). International Society of Sports Nutrition Position Stand: Protein and exercise. J. Int. Soc. Sports Nutr..

[39] Kamei Y., Hatazawa Y., Uchitomi R., Yoshimura R., Miura S. (2020). Regulation of Skeletal Muscle Function by Amino Acids. Nutrients.

[40] Smith R.L., Soeters M.R., Wüst R.C.I., Houtkooper R.H. (2018). Metabolic Flexibility as an Adaptation to Energy Resources and Requirements in Health and Disease. Endocr. Rev..

[41] Srisawat K., Shepherd S.O., Lisboa P.J., Burniston J.G. (2017). A Systematic Review and Meta-Analysis of Proteomics Literature on the Response of Human Skeletal Muscle to Obesity/Type 2 Diabetes Mellitus (T2DM) Versus Exercise Training. Proteomes.

[42] Civitarese A.E., Smith S.R., Ravussin E. (2007). Diet, energy metabolism and mitochondrial biogenesis. Curr. Opin. Clin. Nutr. Metab. Care.

[43] Radak Z., Zhao Z., Koltai E., Ohno H., Atalay M. (2013). Oxygen consumption and usage during physical exercise: The balance between oxidative stress and ROS-dependent adaptive signaling. Antioxid. Redox Signal..

