# Supplementary material for: Metabolic and Transcriptional Adaptations Improve Physical Performance of Zebrafish
Source: Antioxidants (Basel). 2021 Oct 7;10(10):1581. doi: 10.3390/antiox10101581 (PMC8533608; doi:10.3390/antiox10101581)
Supplement: Supplementary file 1 [file antioxidants-10-01581-s001.zip › antioxidants-1394412-supplementary.pdf]

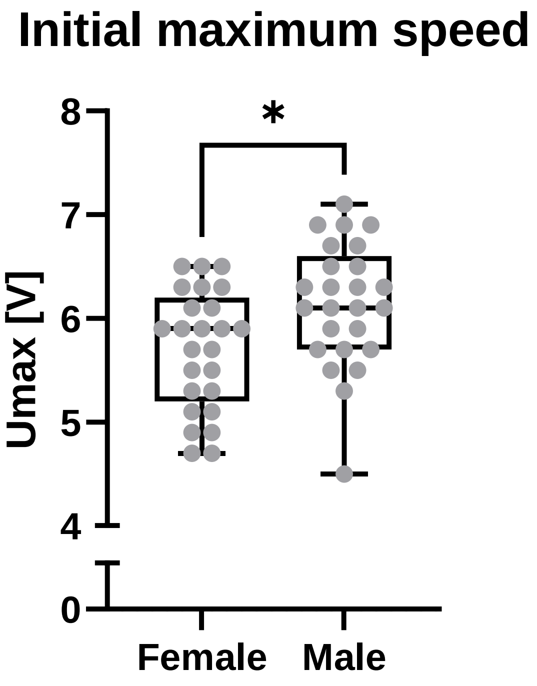

Figure S1: Initial maximum speed was higher in male than in female fish ($p=0.011$). Maximum speed performance was assessed from all fish at the beginning of the experiments. N=25 fish per group. Two-tailed unpaired t-test. *$p<0.05$.

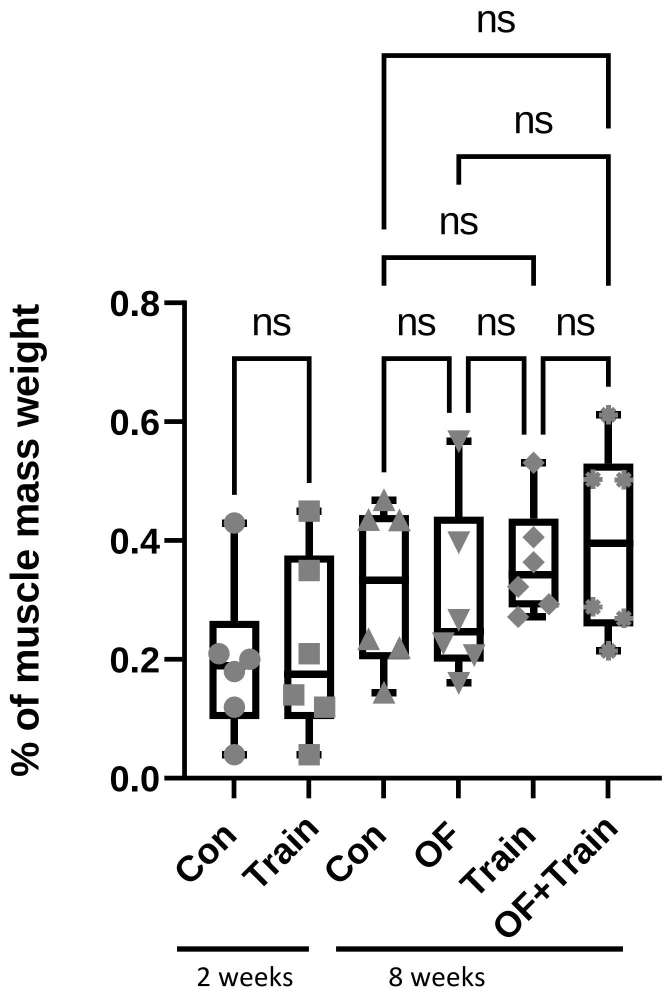

Figure S2: Glycogen content in zebrafish muscle. Glycogen content did not change significantly in muscle after 2 and 8 weeks of training.
